# Genome editing and molecular analyses of an *Arabidopsis* transcription factor, LATE FLOWERING

**DOI:** 10.5511/plantbiotechnology.23.0920a

**Published:** 2023-12-25

**Authors:** Yoshimi Nakano, Maki Kawai, Moeca Arai, Sumire Fujiwara

**Affiliations:** 1 Bioproduction Research Institute, National Institute of Advanced Industrial Science and Technology (AIST), Tsukuba, Ibaraki 305-8566, Japan; 2 Graduate School of Science and Technology, University of Tsukuba, Tsukuba, Ibaraki 305-8572, Japan

**Keywords:** C2H2 zinc finger, EAR motif, flowering, transcriptional repressor

## Abstract

Correct flower organ formation at the right timing is one of the most important strategies for plants to achieve reproductive success. Ectopic overexpression of LATE FLOWERING (LATE) is known to induce late flowering, partly through suppressing expression of the florigen-encoding gene *FLOWERING LOCUS T* (*FT*) in *Arabidopsis*. LATE is one of the C2H2 zinc finger transcription factors, and it has a canonical transcriptional repression domain called the ethylene-responsive element-binding factor-associated amphiphilic repression (EAR) motif at the end of its C terminus. Therefore, LATE is considered a transcriptional repressor, but its molecular function remains unclear. Our genome-edited *late* mutants exhibited no distinct phenotype, even in flowering, indicating the presence of redundancy from other factors. To reveal the molecular function of LATE and factors working with it, we investigated its transcriptional activity and interactions with other proteins. Transactivation activity assay showed that LATE possesses transcriptional repression ability, which appears to be attributable to both the EAR motif and other sequences. Yeast two-hybrid assay showed the EAR motif-mediated interaction of LATE with TOPLESS, a transcriptional corepressor. Moreover, LATE could also interact with CRABS CLAW (CRC), one of the most important regulators of floral meristem determinacy, through sequences in LATE other than the EAR motif. Our findings demonstrated the possibility that LATE can form a transcriptional repression complex with CRC for floral meristem determinacy.

## Introduction

In flowering plants, the phase transition from vegetative to reproductive, or flowering, is one of the most critical events throughout the life cycle for plants to survive and thrive in a fluctuating environment. Both environmental and endogenous cues are involved in the initiation of flowering. One of the most important components mediating the environmental cues is florigen, whose expression is under the control of photoperiodic and other pathways (Amasino 2010). FLOWERING LOCUS T (FT) has been revealed to function as a florigen, which is a mobile small signal molecule. FT protein is expressed in leaves, after which it is translocated from leaves to shoot apical meristem (SAM) through phloem. At the SAM, FT protein forms the florigen complex with FD, a bZIP transcription factor, to induce the expression of floral meristem-identity genes such as *APETALA1* (*AP1*) (Amasino 2010). When plants begin flowering, SAM is transformed into an inflorescence meristem, after which floral meristem appears from the peripheral region of the inflorescence meristem. The floral meristem also contains stem cells. At an early stage of flower development, both proliferation and differentiation of the stem cells are maintained. WUSCHEL (WUS) and AGAMOUS (AG) play pivotal roles in these processes; WUS and CLAVATA3 are required to maintain stem cells and spatially restrict their pool, whereas AG regulates floral determinacy for floral primordium formation. When stage 6 is reached (stages as described by Smyth et al. 1990), floral meristem should be terminated by arresting WUS function for the proper establishment of floral organs (Denay et al. 2017; Sun and Ito 2015; Xu et al. 2019). AG directly induces the expression of *KNUCKLES* (*KNU*), which encodes a C2H2 zinc finger transcription factor, to repress *WUS* expression (Sun et al. 2009). AG directly induces the expression of *CRABS CLAW* (*CRC*), which encodes a YABBY transcription factor, to control floral meristem determinacy. Furthermore, CRC may synergistically regulate *WUS* expression with KNU for floral stem cell termination (Gross et al. 2018; Yamaguchi et al. 2017). This series of processes is regulated by many transcription factors via complex and rigorous mechanisms.

LATE FLOWERING (LATE), a C2H2 zinc finger transcription factor, has been shown to delay the initiation of flowering when ectopically overexpressed (Weingartner et al. 2011). C2H2 zinc finger proteins (ZFPs) form a large family in higher and lower eukaryotes. C2H2 zinc fingers play important roles not only in DNA/RNA binding but also in protein–protein interactions, which is attributed to lineage-specific diversification and expansion (Englbrecht et al. 2004). In the *Arabidopsis* genome, there are 176 ZFPs, which contain one or more zinc finger domains, more than 80% of which are plant-specific. Plant-specific ZFPs are assumed to be involved in transcriptional regulation (Englbrecht et al. 2004). LATE is also a plant-specific ZFP and has one zinc finger domain in the middle of the protein and one canonical repression motif, the ethylene-responsive element-binding factor-associated amphiphilic repression (EAR) motif, at the end of its C terminus. *LATE* is only expressed in the aerial part of seedlings, and its higher expression was observed in the SAM and leaf vasculature (Weingartner et al. 2011). When *LATE* was overexpressed using the CaMV 35S promoter, the plants showed a late flowering phenotype upon shifting conditions from short-day to long-day, in accordance with repression of the *FT*, *SOC1*, and *LEAFY* genes. Moreover, when *LATE* was overexpressed in the vasculature by the promoter of the sucrose-proton symporter gene, plants showed late flowering by suppressing *GIGANTEA* (*GI*), *CONSTANS* (*CO*), and *FT* expression. Meanwhile, when *LATE* was overexpressed by the *FD* promoter in the SAM, plants were defective in floral organ differentiation, resulting in abnormal flowers (Weingartner et al. 2011). According to the phylogenetic analysis, LATE belongs to the C1-1iAa subgroup of C2H2 ZFPs, which contains eight members (Englbrecht et al. 2004). Of these eight members, KNU shares high sequence similarity with LATE. As described above, KNU is involved in floral meristem termination to ensure development of the reproductive organs (Liu et al. 2011). Moreover, *knu* mutant develops abnormal flowers, which have ectopic stamens and carpels (Payne et al. 2004). Given that LATE overexpression in the SAM could induce abnormal flowers, this subgroup might be related to the regulation of floral differentiation. Taking these findings together, LATE has been considered to have dual functions: one affects the establishment and maintenance of floral meristems at the SAM, whereas the other affects the expression of genes involved in the photoperiodic pathway in the vasculature (Weingartner et al. 2011). However, the function of LATE as a transcription factor is still unclear.

To investigate the functions of LATE, we performed genetic and molecular analyses. Our gene-edited *late* mutants showed no distinct phenotype, even in flowering, suggesting redundant factors. By using a transactivation activity assay, we demonstrated that LATE has transcriptional repression ability. In addition, via yeast two-hybrid (Y2H) analysis, we showed that LATE formed homodimers and heterodimers, and constituted a transcriptional repressor complex together with TOPLESS (TPL). Moreover, LATE interacted with CRC through a sequence other than the EAR motif. On the basis of these findings, we propose that LATE may function with CRC as a transcriptional repressor complex to terminate stem cell proliferation and regulate floral organ differentiation. Therefore, LATE overexpression would lead to abnormal flowers.

## Materials and methods

### Plant materials and growth conditions

All wild-type plants used in this study were *Arabidopsis thaliana* accession *Col*-0. Seeds were sown in mixed soil with equal amounts of Supermix A (Sakata Seed) and vermiculite (Asahi Industry), and plants were grown at 22°C under conditions of 16 h light and 8 h dark (long-day) or 8 h light and 16 h dark (short-day). For gene expression analysis, plants were grown on MS medium (0.5% sucrose and 1% agar) at 22°C and light intensity of 120–150 µmol m^−2^ s^−1^ under conditions of 16 h light and 8 h dark. Whole seedlings were collected at zeitgeber time 16, frozen with liquid nitrogen, and then stored at −80°C until further analysis.

### Genome editing

Editing of the *LATE* gene was performed using the CRISPR/Cas9 system. Guide RNA sequences were amplified with primer sets (Supplementary Table S1) and pGTR (Xie et al. 2015) as a template, and then the amplified fragments were cloned into the *Aar*I site of the pKI1.1R vector (Tsutsui and Higashiyama 2017) using Gibson Assembly® Master Mix (New England BioLabs). Using this vector, *Arabidopsis* plants were transformed via *Agrobacterium* in accordance with the floral dip method. The T_1_ generation of transgenic plants was screened on MS medium containing 30 mg l^−1^ hygromycin and 250 mg l^−1^ vancomycin, followed by transfer into the soil. Successful genome editing of the plants was confirmed by PCR and sequence analysis. Those individuals that were homozygous at the editing sites of the *LATE* gene and Cas9-free were selected from the T_3_ progeny. These plants were grown at 22°C under long-day and short-day conditions. Flowering time was quantified by counting the total leaf number when the main inflorescence stem reached to about 5 cm and the number of days after sowing until flower bud formation is confirmed. Statistical analysis comparing WT and genome-edited plants was performed using Student’s *t*-test (*p*<0.05).

### Transactivation activity assay

For vector construction, the full-length *LATE* coding sequence was amplified with the primer set (Supplementary Table S1), after which the amplified fragments were cloned into the pDONR207 vector by the BP reaction (LATE/pDONR207, Thermo Fisher Scientific). Full-length *LATE* and *LATEΔEAR* sequences were amplified with the primer sets and LATE/pDONR207 as the template, and then cloned into the SmaI site of the 430T1.2 vector (Ohta et al. 2000) and p35SVP16 vector harboring the VP16 transcriptional activation domain sequence (Mitsuda et al. 2006) using DNA Ligation Kit (Mighty Mix, Takara). Full-length *LATE* and *LATEΔEAR* sequences on p35SVP16 were amplified with the primer sets, after which VP16-fused full-length *LATE* and *LATEΔEAR* sequences were cloned into the SmaI site of the 430T1.2 vector (Ohta et al. 2000). Full-length *LATE* was amplified with the primer set and LATE/pDONR207 as the template, and then cloned into the SmaI site of the pUBQ1SXG vector harboring the Superman Repression Domain modified ver. X (SRDX) sequence (Yoshida et al. 2013); next, SRDX-fused full-length *LATE* sequence was cloned into the SmaI site of the 430T1.2 vector (Ohta et al. 2000). Mesophyll protoplasts were isolated from 4-week-old *Arabidopsis* leaves as described previously (Sakamoto et al. 2016). Each effector plasmid was transiently co-transfected in the protoplast using the PEG/Ca^2+^ method with a reporter plasmid harboring the firefly luciferase (*f*LUC) gene under the control of 5×Gal4 DNA binding sites and a reference plasmid harboring the codon-optimized luciferase gene from *Renilla* (*r*LUC) under the control of the 35S promoter. Blank vector of 430T1.2 was used as a control. After 16 h of incubation at 22°C in the dark, the protoplasts were treated with Dual-luciferase reporter assay system (Promega) and the luminescence was analyzed by Infinite F200 (TECAN). The relative transactivation activities were calculated by dividing the reporter activity by the reference activity. Statistical analysis was performed by pairwise *t*-test with Holm correction by R software. Differences were considered statistically significant at *p*<0.05.

### Yeast two-hybrid assay

For a bait plasmid, the *LATE* and *LATEΔEAR* sequences in the pDONR207 vector were transferred into the pDEST_GBKT7 vector harboring the GATEWAY cassette (*att*R1-*ccdB*/Cm^r^-*att*R2) between GAL4 DNA-BD and the ADH1 terminator of pGBKT7 (Chrontech) by the LR reaction (Thermo Fisher Scientific). These bait vectors were transformed into yeast strain Y2H gold and selected using SD medium lacking tryptophan (-W). Positive clones were confirmed by PCR using a primer set (Supplementary Table S1). Full-length coding sequences of *KNU*, *CRC*, *TPL*, and *HDA19* were amplified by PCR with specific primer sets (Supplementary Table S1) and cloned into pDONR207 by the BP reaction (Thermo Fisher Scientific). For prey plasmids, *KNU*, *CRC*, *TPL*, and *HDA19* sequences in the pDONR207 vector were transferred into the pDEST_GADT7 vector by the LR reaction (Thermo Fisher Scientific). These prey plasmids or blank control plasmid (pDEST_GADT7) were transformed into the yeast harboring the bait plasmids and spotted onto SD medium lacking tryptophan and leucine (-W/-L) or SD medium lacking tryptophan, leucine, and histidine (-W/-L/-H) supplemented with 0, 0.05, or 0.1 mM 3-amino-1,2,4-triazole (3-AT). These yeasts were grown at 30°C in the dark for 7–10 days.

## Results

### LATE protein possesses a transcriptional repression ability

**Figure 1:**
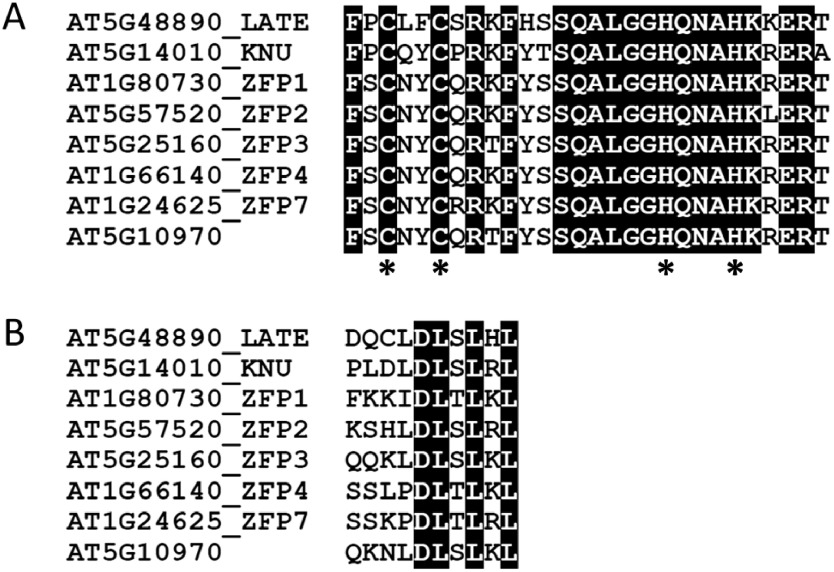
Figure 1. Amino acid sequence comparisons of C2H2 ZFPs among the C1-1iAa subgroup. (A) Amino acid alignment of C2H2 zinc finger domain. Asterisks indicate cysteine (C) and histidine (H), which contribute to binding zinc ions. (B) Amino acid alignment of EAR motif. The consensus sequence of either “LxLxL” or “DLNxxP” is located at the C-terminal of the proteins. Residues conserved in all eight proteins are shaded.

LATE belongs to the C1-1iAa subgroup of C2H2 ZFPs, which includes eight members (Englbrecht et al. 2004), the sequences of which we initially compared. All eight members have one C2H2 zinc finger domain (Figure 1A). In addition, an EAR transcriptional repression motif, whose consensus sequence is either “LxLxL” or “DLNxxP”, is found at the C-terminal end of these members (Figure 1B). Accordingly, all members of this subgroup are assumed to be transcriptional repressors, and thus LATE might be involved as a repressor in the transcriptional regulation of flowering and/or floral meristem differentiation.

**Figure 2:**
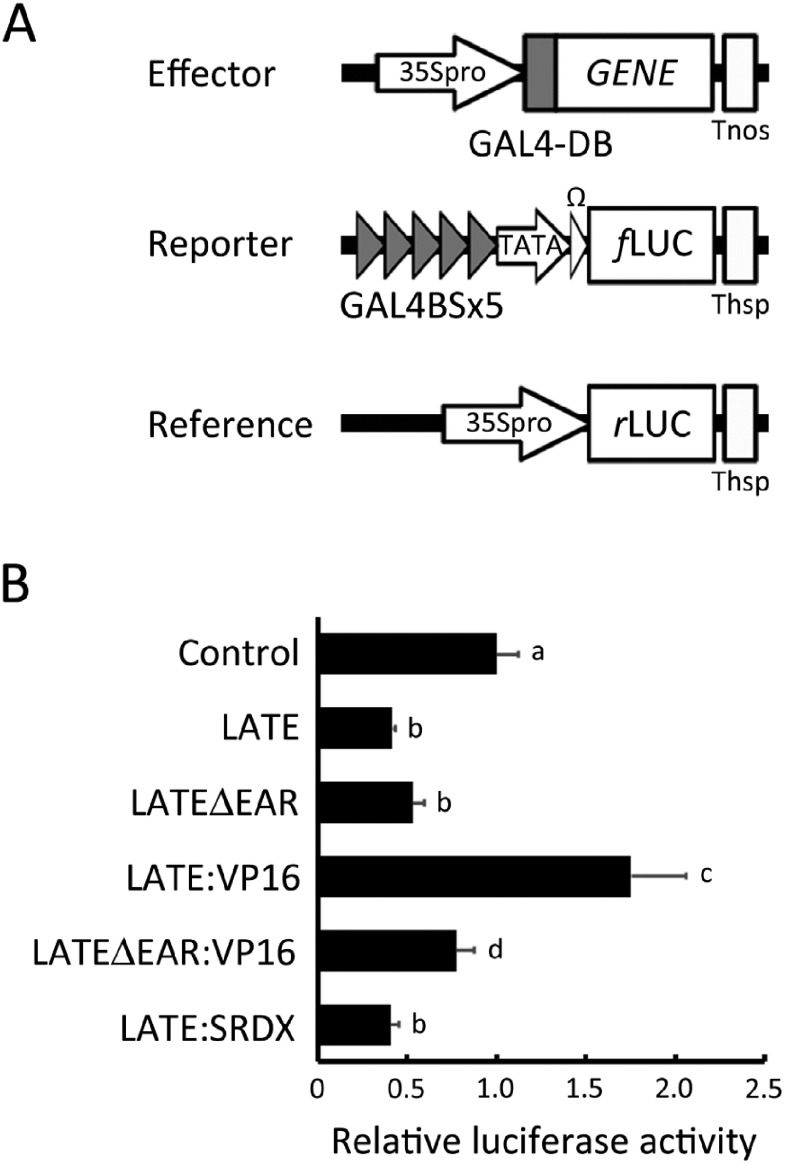
Figure 2. Transactivation activity assay of LATE. (A) A schematic drawing of the plasmids. Effector plasmids containing the Gal4-DB-fused full-length coding sequence of LATE, LATEΔEAR, LATEΔEAR:VP16, LATE:VP16, or LATE:SRDX were cotransfected into protoplasts derived from *Arabidopsis* leaves with the reporter plasmid containing *f*LUC driven by 5×Gal4-binding sites and the reference plasmid containing *r*LUC. (B) Transactivation activities of LATE proteins. Transactivation activities were calculated by dividing *f*LUC activity by *r*LUC activity, and the activity of the control was set as 1. Bars indicate standard deviation of eight replicates. Bars with different letters a, b, and c are significantly different from each other (pairwise *t*-test with Holm correction, *p*<0.05).

To clarify the transcriptional ability of LATE protein, we conducted a transactivation activity assay by transiently overexpressing proteins fused with Gal4 DNA-binding domain in *Arabidopsis* mesophyll protoplasts. We found a highly conserved EAR motif at the C-terminal end of LATE protein (Figure 1B), and thus we investigated whether this motif affects the transcriptional ability of this protein. Effector plasmids containing sequences encoding full-length LATE, LATE without the EAR motif (LATEΔEAR), VP16-fused LATE (LATE:VP16), VP16-fused LATEΔEAR (LATEΔEAR:VP16), and SRDX-domain-fused LATE (LATE:SRDX) were cotransfected with reporter and reference plasmids into protoplasts prepared from *Arabidopsis* leaves (Figure 2A). LATE protein showed significantly low transactivation activity compared with the control (empty vector), and the activity was 60% less than that of the control (Figure 2B), which suggested that LATE possesses transcriptional repression ability. When the EAR motif located at the C terminus was removed, the transactivation activity of LATEΔEAR was not significantly but slightly increased compared with that of LATE, and it was still significantly lower than that of the control. This indicated that the transcriptional repression ability of LATE might be partially attributable to the EAR motif, and other parts of the LATE protein still possess repression ability. When the VP16 activation domain was fused with the LATE protein, its transactivation activity was increased more than 1.75-fold. Meanwhile, the transactivation activity reached more than 80-fold when Gal4 DNA-binding domain fused with only VP16 was overexpressed (Supplementary Figure S1), suggesting the possibility that VP16 transactivation activity was suppressed by LATE. Unexpectedly, in the case of LATEΔEAR:VP16, the effect of VP16 on transactivation was less apparent comparing to the case of LATE:VP16, and its transactivation activity was lower than that of the control. Why LATEΔEAR:VP16 showed less transactivation activity is presently unclear. The transactivation activity of LATEΔEAR:VP16 was indeed significantly higher than that of LATEΔEAR, showing a slight activation effect by VP16. The transactivation activity of LATE:SRDX was comparable to that of LATE. SRDX is a repression domain consisting of only 12 amino acids, which resembles the EAR motif, and actively converts transcriptional activators into repressors in plant cells (Hiratsu et al. 2003; Mitsuda et al. 2011). This indicates that tandemly repeated repression domains cannot enhance the repression ability of the LATE protein. These findings suggest that LATE has a transcriptional repression ability, which could potentially be attributed to the EAR motif at the end of its C terminus, along with other yet-to-be-identified domain(s).

### Plants with genome editing of *LATE* do not show early flowering phenotype

**Figure 3:**
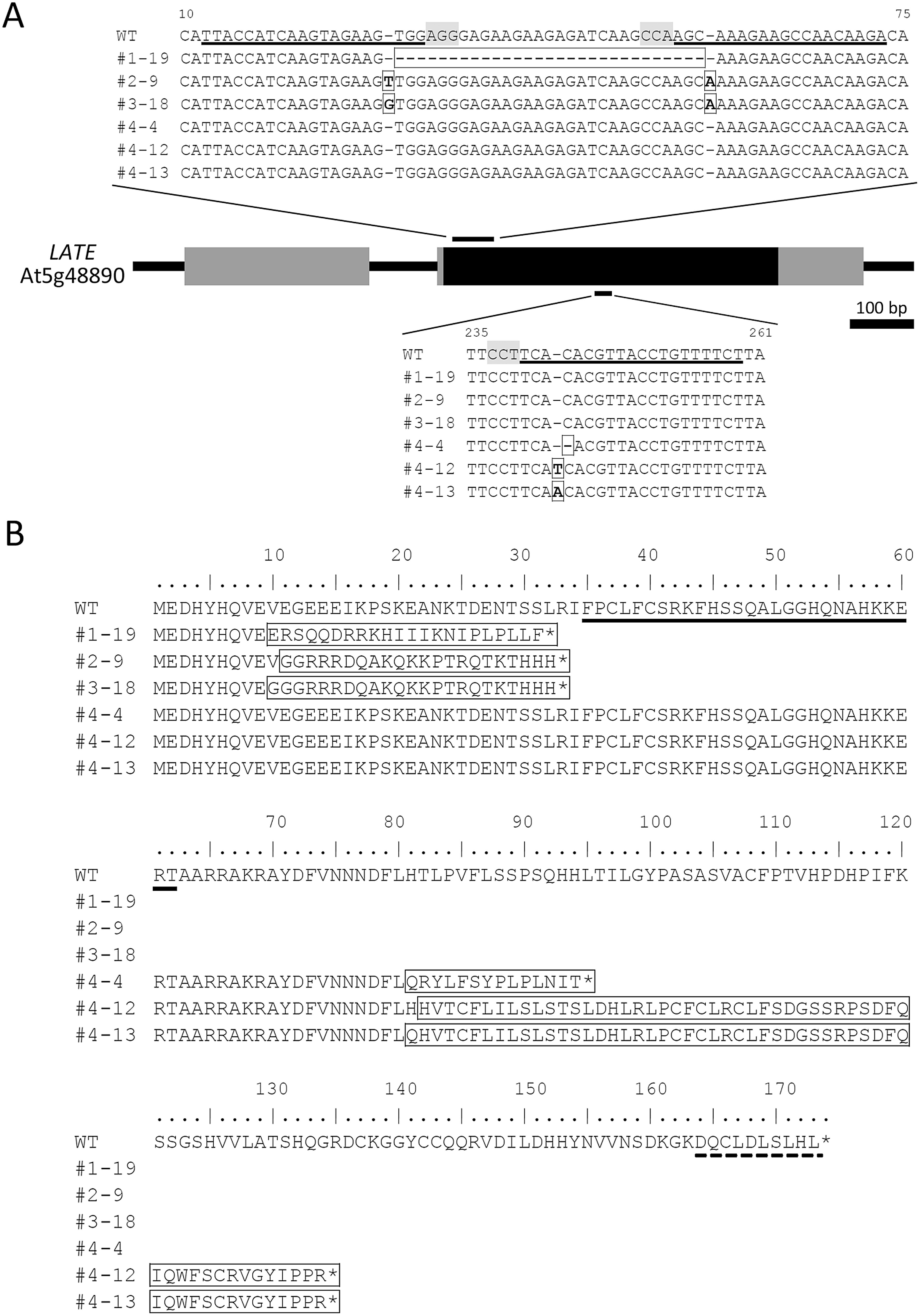
Figure 3. Genome editing of the *LATE* gene using CRISPR-Cas9 in *Arabidopsis*. (A) A schematic drawing of the structure of the *LATE* gene (gray boxes, UTRs; black boxes, CDS) and the sites of genome editing. PAM sequences are shaded and followed by gRNA sequences designed in this study (underlined). Each insertion/deletion of genome-edited lines is framed. (B) Amino acid alignment of LATE proteins predicted from DNA sequences of the WT and genome-edited plants. C2H2 Znf domain and EAR motif are underlined by solid and dashed lines, respectively. Each amino acid altered by genome editing of the genome-edited lines is framed.

**Figure 4:**
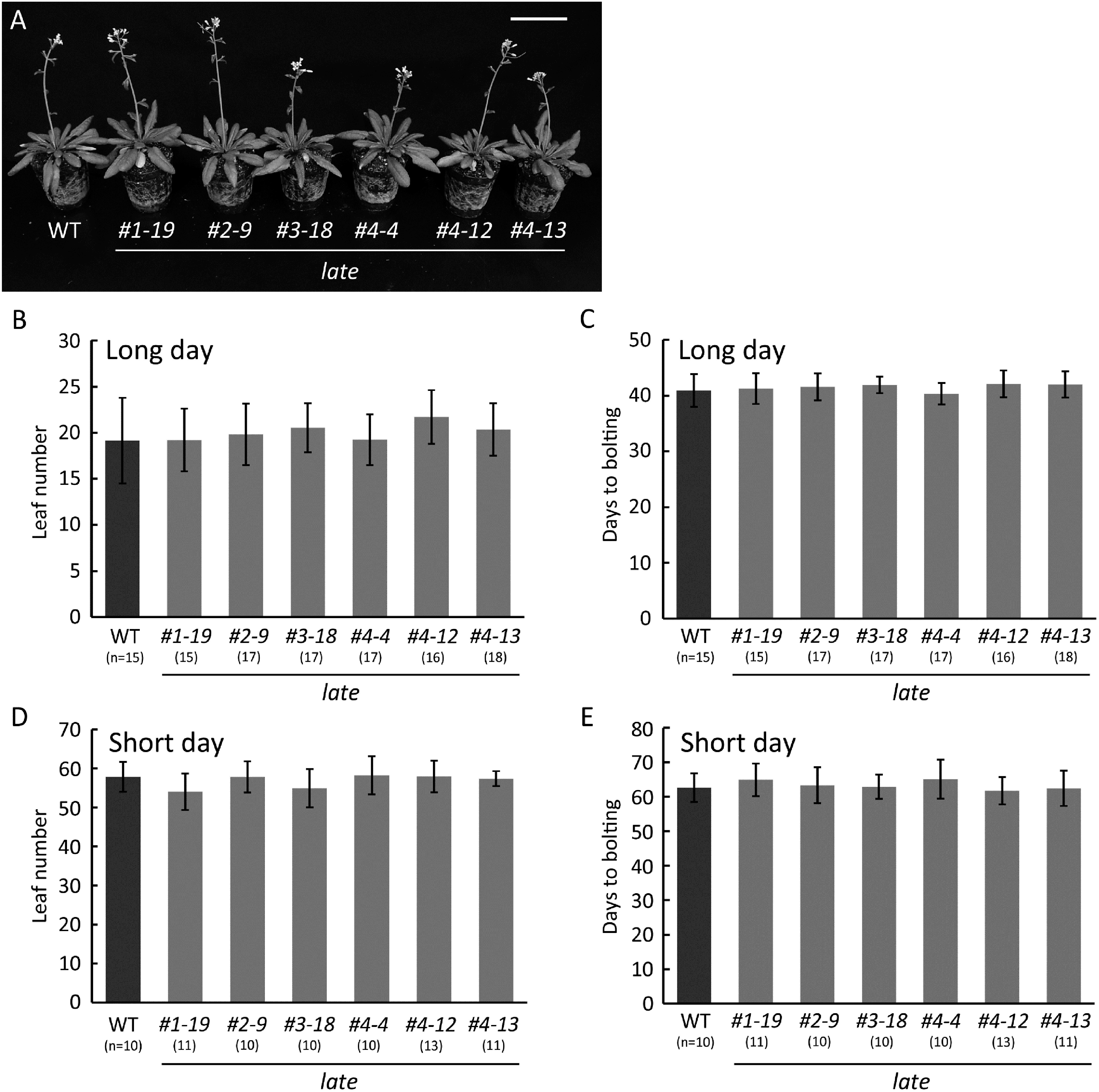
Figure 4. Genome-edited plants and flowering time. (A) Six-week-old plants of the WT and genome-edited lines grown on soil at 22°C under long-day conditions (16 h light and 8 h dark). Bar=5 cm. (B and C) Total leaf number (B) and days to bolting (C) of the WT and genome-edited plants grown on soil at 22°C under long-day conditions (16 h light and 8 h dark). (D and E) Total leaf number (D) and days to bolting (E) of the WT and genome-edited plants grown on soil at 22°C under short-day conditions (8 h light and 16 h dark). The numbers listed under the plants demonstrate the numbers of plants tested. Error bars indicate the standard deviation. No significant differences between the WT and genome-edited plants were observed (*t*-test, *p*<0.05).

Overexpression of LATE was reported to induce the late flowering phenotype and reduce the expression of genes involved in flowering (Weingartner et al. 2011). Therefore, we thought that the lack of LATE would lead to upregulation of these genes and plants would exhibit early flowering. To investigate the impact of the defect of the *LATE* gene on flowering, we generated *late* mutants by genome editing because no T-DNA insertion line for the *LATE* gene was available. The coding sequence of *LATE* is composed of only one exon of 522 bp (including the stop codon), and thus the LATE protein is deduced to contain 173 amino acids. We designed four gRNA sequences and set two combinations for genome editing. One combination was targeted to sites close to the start codon and the other was targeted to the middle sites of the *LATE* gene (Figure 3A). We obtained six *late* mutant alleles, with the editing occurring at three positions. Each mutation of the mutants was as follows: a 29-bp deletion in line *1-19*, two 1-bp insertions in lines *2-9* and *3-18*, a 1-bp deletion in line *4-4*, and a 1-bp insertion in lines *4-12* and *4-13* (Figure 3A). All of these presumably lacked the full-length LATE protein due to premature stop codons, and for lines *1-19*, *2-9*, and *3-18* even the zinc finger domain was lost, indicating that these mutants are null alleles (Figure 3B). The transcript levels of the *LATE* gene in some lines were slightly and not significantly decreased (Supplementary Figure S2), which may have been attributable to small indels in the sequence. All mutant lines were viable and fertile (Figure 4A). Contrary to expectations, the flowering time of *late* mutants was comparable to that of the WT both under long-day and short-day conditions (Figure 4B–E), indicating that LATE and unknown protein(s) may function redundantly in the regulation of flowering.

### LATE formed homo- and heterodimers

Given that *late* mutants did not show the early flowering phenotype, we assumed that some proteins could function redundantly with LATE for the regulation of flowering. Therefore, to investigate the interaction of LATE with other proteins, yeast two-hybrid assay was performed. We generated two types of bait plasmids: LATE and LATEΔEAR. For prey plasmids, we selected five proteins, including LATE. We did not find any homologous gene of LATE in the *Arabidopsis* genome, while, of the eight members of the C1-1iAa subgroup of ZFPs, KNU shares the highest sequence similarity with LATE. Moreover, KNU is involved in the regulation of floral meristem determinacy, which raised the possibility that LATE might interact with KNU, after which they function together at the SAM. An EAR motif is also conserved in KNU at the end of its C terminus (Figure 1B), and through this motif, KNU can interact with TPL and HDA19 to form a transcriptional repressor complex (Bollier et al. 2018). A large-scale interactome analysis previously revealed the protein–protein interaction between LATE and CRC (Wanamaker et al. 2017); moreover, CRC was also shown to be involved in the regulation of floral meristem determinacy in parallel with KNU (Sun and Ito 2015; Xu et al. 2019). Given the above findings, we decided to confirm the interaction of LATE with CRC.

**Figure 5:**
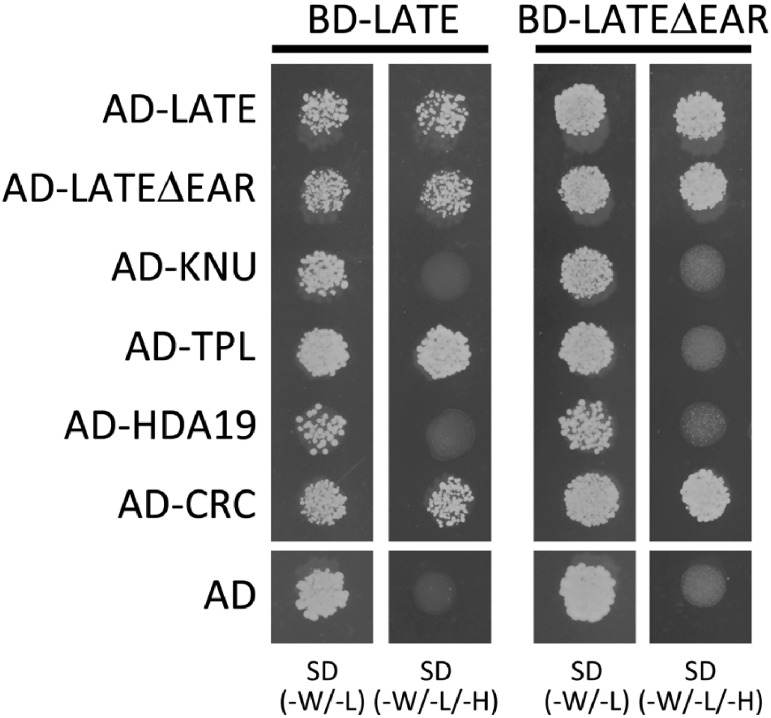
Figure 5. Protein–protein interactions by yeast two-hybrid assay. Bait plasmids of either LATE or LATEΔEAR fused with the Gal4 binding domain (BD) were cotransformed into yeast with prey plasmids of LATE, LATEΔEAR, KNU, TPL, HDA19, or CRC fused with the Gal4 activation domain (AD) or blank plasmid containing only AD; then, these yeasts were spotted onto the medium SD (-W/-L) or SD (-W/-L/-H).

The results of this study confirmed the interaction of LATE with CRC. In addition, full-length LATE could interact with full-length LATE, which indicated that LATE could form a homodimer (Figure 5). Moreover, full-length LATE could interact with LATEΔEAR, TPL, and CRC. In the case of LATEΔEAR as the bait, interactions were observed between LATEΔEAR and full-length LATE, LATEΔEAR, and CRC, while no interaction was observed between LATEΔEAR and TPL. These results indicated that LATE could interact with LATE and CRC through a sequence other than the EAR motif, while the EAR motif might be necessary for the interaction with TPL. This in turn indicated that LATE could bind to TPL through its EAR motif and form a transcriptional repressor complex. Because no interaction was observed between LATE and HDA19, LATE could not directly bind HDA19 and TPL may serve as the bridge between them. These findings suggest the possibility that LATE could form a transcriptional repressor complex with CRC and TPL at the SAM and influence the regulation of floral meristem determinacy.

## Discussion

In this study, we demonstrated the possibility that LATE could regulate floral organ formation as a member of the transcriptional repressor complex containing CRC and TPL. LATE is one of the C2H2 ZFPs and has an EAR motif at the end of its C terminus (Figure 1). In the Gal4-binding system, both LATE and LATEΔEAR proteins showed the ability to repress transcription (Figure 2). The EAR motif, whose consensus sequence is either LxLxL or DLNxxP, is a major active repression motif in plants; moreover, several other sequences have been demonstrated to be active repression motifs (Kagale and Rozwadowski 2011). Although we could not find any distinct sequences of known repression motifs other than the EAR in LATE, the repression ability of LATE appears to be attributable to not only the EAR motif but also other sequences. This ability was sufficient to lessen the activation activity of VP16. Fusion of the chimeric repression domain, SRDX, to a transcriptional activator, is known to convert it to a repressor. On the other hand, it either enhances the repression activity or has no effect when fused to a transcriptional repressor (Mitsuda et al. 2011). Fusion of the SRDX did not show any significant effect on the repression activity of LATE (Figure 2). Taking these findings together, we concluded that LATE could function as a transcriptional repressor. When the VP16 domain was fused with LATEΔEAR, its transactivation activity was lower than that of LATE:VP16, contrary to expectations. This data implies that the EAR of LATE not only functions as a repression motif but also is necessary for its role as a transcriptional regulator. Conducting further domain analysis on LATE would provide more insights. Given that EAR deletion did not show significant effect on the transactivation activity of LATE (Figure 2), not only the EAR but other parts of the LATE protein would also possess the repression ability. In the transient reporter assay system used in this study, the absence of interacting factors necessary for the intrinsic function of LATE could have affected the results. Further analysis using other experimental systems would provide more insights.

In yeast, the EAR motif was shown to be needed for the interaction of LATE with TPL, which indicated that LATE would form a protein complex with TPL as a transcriptional repressor. Our results also demonstrated that LATE could form a homodimer and that a part of LATE other than the EAR motif was needed for interaction with CRC. Meanwhile, no interaction of LATE with KNU, which has the sequence most similar to LATE, was observed (Figure 5). KNU regulates floral meristem differentiation in a manner controlled by AG. Mutation of *KNU* was reported to lead to abnormal flowers with enlarged carpels and ectopic stamens, and KNU was found to negatively regulate the cell proliferation of floral meristem (Payne et al. 2004). CRC is also directly regulated by AG and involved in floral meristem determinacy as a transcriptional repressor (Gross et al. 2018; Yamaguchi et al. 2017). KNU and CRC synergistically regulate *WUS* expression, which is needed for floral stem cell termination, and such regulation is needed to ensure the proper development and determinacy of reproductive organs (Sun and Ito 2015; Xu et al. 2019). Our results raised the possibility that LATE could form a protein complex with CRC, not KNU. CRC is one of the members of the YABBY transcription factor family, which has six members. CRC has both one zinc finger domain and one EAR motif, the same as LATE. What sequences in the LATE protein would be needed for the interaction with CRC and whether LATE could form a protein complex with CRC to regulate *WUS* expression, in contrast to KNU, would be interesting to investigate. In this study, we only analyzed the relationship between LATE and CRC, and thus the relationships between LATE and other YABBY proteins remain to be elucidated, given that all members are expressed in flowers (Bowman and Smyth 1999; Siegfried et al. 1999; Villanueva et al. 1999). Although *late* mutants did not show any apparent defect in flower formation, more detailed morphological analysis of flower development is needed. In addition, double or multiple mutants of LATE and YABBY proteins including CRC, KNU and other subfamily members, and TPL family proteins, may provide clues for understanding the relationship between LATE and other proteins for flower organ formation.

LATE-overexpressing plants showed late flowering and formed abnormal flowers (Weingartner et al. 2011). Accordingly, we hypothesized that mutants lacking LATE would show an early flowering phenotype. We created six *late* mutants by genome editing and examined their flowering time. Our results showed that the *late* mutants did not exhibit an early flowering phenotype or any significant deficiency in growth (Figure 4). We grew *late* mutants under the conditions described in Weingartner et al. (2011) as LATE-overexpressing plants showing late flowering, but the flowering time of *late* mutants was comparable to that of the WT plants. Thus, the early flowering phenotype might emerge in very limited conditions or might involve the functional paralog(s) of LATE. Analysis of double or multiple mutants of *late* with potential redundant genes would be needed for further understanding the function of LATE in the flowering time regulation. We obtained six independent genome edited lines of *late*; lines *1-19*, *2-9*, and *3-18* lacked the zinc finger domain, while lines *4-4*, *4-12*, and *4-13* retained this domain (Figure 3). Given that the zinc finger domain remained in the latter three lines (Figure 3), the truncated forms of the LATE protein might have bound to the target sequences; however, they would act aberrantly due to the lack of the C-terminal half of the protein, including the EAR motif. These truncated forms of LATE might not repress the target genes because of the lack of the EAR motif, which is necessary to bind TPL. Whether these truncated forms of LATE can interact with CRC and possess transcriptional repression ability would be interesting to investigate.

In summary, our results shed light on the possible role of LATE in the regulation of floral organ determinacy, and further analyses of *late* mutants and the transcriptional repression complex containing LATE may provide clues for understanding the involvement of LATE in the regulation of flowering.

## Abbreviations

3-AT: 3-amino-1,2,4-triazole
AG: AGAMOUS
AP1: APETALA1
CO: CONSTANS
CRC: CRABS CLAW
EAR: ethylene-responsive element-binding factor-associated amphiphilic repression
FT: FLOWERING LOCUS T
GI: GIGANTEA
KNU: KNUCKLES
LATE: LATE FLOWERING
SAM: shoot apical meristem
SRDX: Superman Repression Domain modified ver. X
TPL: TOPLESS
WUS: WUSCHEL
Y2H: yeast two-hybrid
ZFP: zinc finger protein

## Description of Supplementary Files

Supplementary Figure S1. Transactivation activity assay of VP16, SRDX, and LATE.

Supplementary Figure S2. Semiquantitative RT-PCR analysis of *LATE* gene expression of the genome editing plants.

Supplementary Table S1. Primer list for this experiment.
