# Supplementary material for: Genome editing and molecular analyses of an Arabidopsis transcription factor, LATE FLOWERING
Source: Plant Biotechnol (Tokyo). 2023 Dec 25;40(4):337–44. doi: 10.5511/plantbiotechnology.23.0920a (PMC10905564; doi:10.5511/plantbiotechnology.23.0920a)
Supplement: Supplementary Data [file plantbiotechnology-40-4-23.0920a-s001.pdf]

Supplemental data

Nakano and Kawai et al.

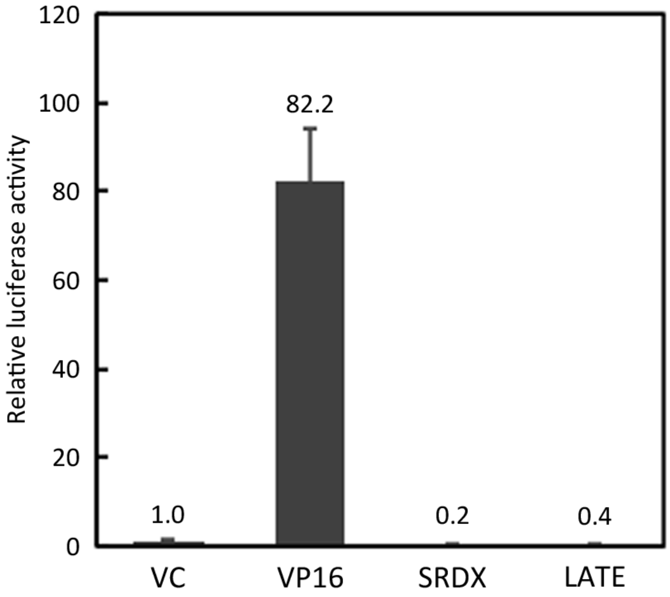

Supplementary Figure S1. Transactivation activity assay of VP16, SRDX, and LATE.

VP16 domain, SRDX domain, or LATE were fused with Gal4-DB and cotransfected into protoplasts derived from Arabidopsis leaves with the reporter plasmid harboring the *f*LUC driven by 5 × Gal4-binding sites and a reference plasmid harboring the *r*LUC. The relative transactivation activities were calculated by dividing the reporter activity by the reference activity, and the activity of vector control was set to 1. Bars indicate standard deviation of eight replicates. The numbers at the top of each bar indicate the relative luciferase activity for each sample.

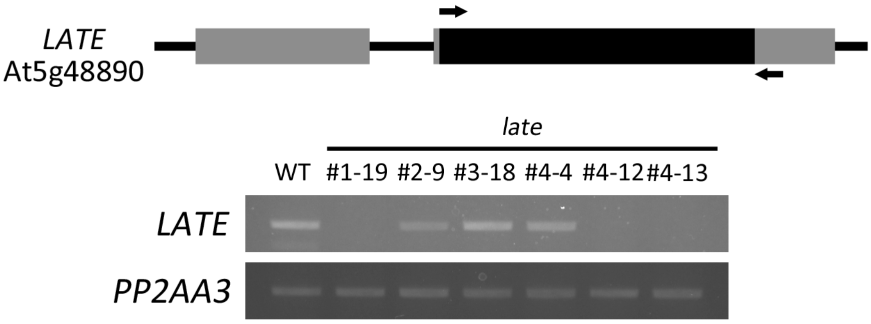

Supplementary Figure S2. Semiquantitative RT-PCR analysis of *LATE* gene expression of the genome editing plants.

Total RNA was purified from seedlings seven days after germination. Total RNA extraction, DNase treatment, and reverse transcription were performed as described previously (Nakano et al. 2017 Plant Sci). cDNA was appropriately diluted and used for semi-quantitative RT-PCR using Ex Taq (TAKARA) and specific primer sets (Supplemental Table). *PP2AA3* was used as the internal control. A schematic drawing shows the structure of *LATE* gene (gray boxes, UTRs; black boxes, CDS) and arrows indicate the sites where the primers were designed.
